# Psycho-Socioeconomic bio-behavioral influences on health-related quality of life

**DOI:** 10.15171/hpp.2017.23

**Published:** 2017-06-14

**Authors:** Meghan K. Edwards, Paul D. Loprinzi

**Affiliations:** Department of Health, Exercise Science and Recreation Management, Physical Activity Epidemiology Laboratory, Exercise Psychology Laboratory, The University of Mississippi, University, MS, USA

**Keywords:** Epidemiology, Health, NHANES, Quality of life

## Abstract

**Background: ** The cumulative effects of psychological, socioeconomic, biological and behavioral parameters on health-related quality of life (HRQOL) has not been thoroughly evaluated, which was this study’s purpose.

**Methods:** Data from the 2005-2006 National Health and Nutrition Examination Survey were used (N = 2524 adults 20-85 years). HRQOL assessed from the Centers for Disease Control and Prevention HRQOL survey, with a higher score indicating worse HRQOL. Evaluated parameters included psychological function, socioeconomic risk, biological function and behavioral parameters. Ultimately, 14 psycho-socioeconomic bio-behavioral (PSBB) parameters were evaluated.

**Results: ** For every 1 unit increase in the PSBB index, participants had a corresponding -0.35 lower HRQOL score (β =-0.35; 95% CI: -0.44, -0.25; P<0.001). All individual components of PSBB were inversely associated with PSBB.

**Conclusion:** PSBB was both individually and cumulatively associated with HRQOL.

## Introduction

Emerging work highlights the importance of considering psychological, socioeconomic, biological and behavioral factors as indicators of health status.^1^ Taken together, recent work has considered each of these four parameters individually and collectively in predicting mortality risk.^1^ Most markedly, adults in the United States who had a higher (reflecting better) psycho-socioeconomic bio-behavioral (PSBB) index score had a reduced mortality risk, and when examined individually, behavioral and socioeconomic parameters were also predictive of mortality risk.^1^ Here, we evaluate this paradigm while considering health-related quality of life (HRQOL) as the outcome of interest.

Notably, HRQOL has been defined as a multi-dimensional construct including physical, mental, emotional, and social functioning components, one which extends beyond direct measures of population health, life expectancy, and causes of death to focus on the influence *health status* has on one’s quality of life.^2^ Evaluating PSBB as it associates with HRQOL is a worthwhile investigation, as it allows for an examination of the potential combined associations between multiple factors that may contribute to one’s HRQOL, as opposed to isolating only one predictor variable. A significant association between PSBB scores and HRQOL is plausible, given previous work demonstrating associations between the individual PSBB components and HRQOL. For instance, Gaynes and colleagues^3^ found the deleterious effects of depression on HRQOL to be comparable to those of diabetes, hypertension, and arthritis, advocating that (in alignment with the present research investigation) a multidimensional approach to promoting ideal HRQOL is pursued as opposed to a one-dimensional treatment strategy. With regards to socioeconomic status (SES), there is considerable evidence establishing the direct association between SES and HRQOL, including studies that have observed a significant association even when controlling for major disease risk factors and chronic illnesses (e.g., obesity).^4,5^ Other work has evaluated biological risk factors such as obesity^6^ and diabetes,^7^ demonstrating deleterious associations with HRQOL. To illustrate an example of the contributing evidence for behavioral factors, Vogl and colleagues^8^ present evidence of a significant, maladaptive association between smoking-status (i.e., smoker, ex-smoker, never smoker) and HRQOL, also supporting an interrelation of the various HRQOL domains. For instance, heavy smokers (as compared to never smokers), were significantly more likely to report “some or severe problems” with regards to mobility, self-care, activity, pain/discomfort, and anxiety/depression.

Although, of course, longevity is of individual and public health importance, enhancing quality of life is also of great importance. Perceived quality of life and longevity may, together, provide a comprehensive assessment of overall health. As such, and taking into account the aforementioned evidence demonstrating the ability for HRQOL to be influenced by a variety of factors, the purpose of this study was to evaluate the association between PSBB scores (including socioeconomic, biological and behavioral factors) and HRQOL, among a national sample of adults.

## Materials and Methods

### Study design and participants

Details on the employed study design and evaluated parameters can be found elsewhere.^1^ Briefly, data were used from the 2005-2006 National Health and Nutrition Examination Survey (NHANES). This NHANES cycle was evaluated as this is the only NHANES cycle that included all PSBB parameters assessed herein. Procedures were approved by the NCHS review board; consent obtained prior to data collection. 2524 participants (20-85 years) provided data on the study variables. The NHANES is an ongoing survey conducted by the Centers for Disease Control and Prevention (CDC) that uses a representative sample of non-institutionalized US civilians selected by a complex, multistage, stratified, clustered probability design.

### Measurement of HRQOL

Measurement of the CDC HRQOL involves 4 questions, with details described elsewhere.^1^

An overall HRQOL score was computed, ranging from 0-4, with higher scores indicative of unfavorable HRQOL.

### Measurement of Psycho-Socioeconomic Bio-Behavioral Influences

*Psychological.* As thoroughly described elsewhere,^1^ the psychological parameter assessed herein was depression symptomology, via the PHQ-9. Those with a PHQ-9 score of 10 or higher were considered herein to have moderate or greater depression symptomology.

*Socioeconomic.* As thoroughly described elsewhere,^1^ SES was determined from four parameters, including poverty level, education, minority status, and social living status.

*Biological.* As thoroughly described elsewhere,^1^ biological parameters included herein were cholesterol, weight status, diabetes, hypertension and systemic inflammation.

*Behavioral.* As thoroughly described elsewhere,^1^ behavioral parameters assessed herein included physical activity (accelerometry), dietary behavior (self-report), smoking status (cotinine) and sleep (self-report).

### Calculation of a psycho-socioeconomic bio-behavioral index score

As thoroughly described elsewhere,^1^ 14 PSBB parameters were assessed; thus, our computed PSBB score ranged from 0-14. As thoroughly described elsewhere,^1^ each of PSSB parameters were coded as 0 or 1, with “1” indicating a favorable score.

### Statistical analyses

Statistical analyses were performed in Stata (v. 12, College Station, TX, USA). All analyses included the utilization of survey sample weights. Multivariable ordinal regression models were used to examine the association between PSBB index and HRQOL. Covariates included age (years; continuous) and gender. Statistical significance established as *P *< 0.05.

## Results

Characteristics of the study variables are shown in Table 1. Participants, on average, were 46.2 years of age and the sample was equally distributed across gender (50.8% female). Table 2  displays the weighted multivariable ordinal regression results evaluating the associations between PSBB and HRQOL. The PSBB index was both individually and cumulatively associated with HRQOL. For every 1 unit increase in the PSBB index, participants had a corresponding -0.35 lower HRQOL score (*P*<0.001). Notably, as identified previously, a higher PSBB and a lower HRQOL are favorable, so the observed inverse association between PSBB and HRQOL is a favorable association. In addition to the observed cumulative association of PSBB on HRQOL, all individual components of PSBB were inversely associated with PSBB. Results were similar when evaluating the association between the index score and the individual components of HRQOL. After adjustments, and for a 1 unit increase score in the index variable, participants had a 38% reduced odds of having fair/poor health (odds ratio [OR]=0.68; 95% CI: 0.56-0.68; *P*<0.001), a 22% reduced odds of having poor physical health (OR=0.78; 95% CI: 0.70-0.88; *P*=0.001), a 23% reduced odds of having poor mental health (OR=0.77; 95% CI: 0.71-0.85; *P*<0.001), and a 39% reduced odds of their physical or mental health keeping them from engaging in usual activities (OR=0.71; 95% CI: 0.63-0.79; *P*<0.001).

## Discussion

Given evidence of psychological, socioeconomic, biological, and behavioral factors associating with mortality risk,^9-12^ previous emergent work^1^developed an index variable, PSBB, to examine the combined associations of these factors on the outcome of all-cause mortality. The original investigation of this novel paradigm demonstrated an inverse association between PSBB and all-cause mortality (i.e., a higher PSBB score was associated with a reduced risk of early mortality).^1^ Notably, this previous study^1^ also demonstrated that the behavioral and socioeconomic index parameters were independently predictive of mortality risk. Considering evidence supporting associations of each of the four PSBB components with HRQOL, and the importance of promoting not only a long life (i.e., preventing premature mortality) but also a life of high quality, we were interested in extending this paradigm to the outcome of HRQOL. The main finding of the present investigation was that PSBB was favorably (inversely) associated with HRQOL, indicating a higher PSBB score is associated with more optimal HRQOL. A notable difference between this study and the previous exploration of PSBB and morality, however, is that in the present investigation *all* individual PSSB components were inversely associated with HRQOL (as opposed to only behavioral and sociodemographic factors). It is difficult to explain these discrepant findings. These observations collectively seem to suggest that psychological, socioeconomic, biological and behavioral factors may all play a critical role in *current* perceived health status, but in the long-term and when considering prevention of premature mortality, some health-related factors may play a larger role in influencing survival.

Ultimately, the current findings underscore the importance of promoting health-enhancing behaviors (e.g., regular exercise, healthy diet, smoking avoidance, and adequate sleep) to positively influence biological functioning (e.g., reduced cardiovascular disease risk)^13-19^ and psychological well-being,^20-22^ all of which may positively influence one’s quality of life. Consistent monitoring of biological health status (e.g., assessment of cardiorespiratory fitness at an annual physical doctor appointment) is of critical importance, as it may help to identify risks for future health problems and may even serve as a catalyst for future behavior change.^23,24^ Additionally, given the observed independent association of the socioeconomic index variable on HRQOL, promotion of these health-enhancing behaviors among vulnerable populations (e.g., minorities, those living below the poverty level) is of major public health interest. This, undoubtedly, is a challenging task given the observed interaction effects of SES on behavioral, biological and psychological outcomes.^25,26^

Major strengths of this study include the comprehensive assessment of PSBB, evaluating objective measures of individual components of the PSBB, employing a national sample, evaluating a novel paradigm, and extending the previous PSBB-mortality work. The findings of this study should, however, be interpreted in the context of the study’s limitations. Mostly notably is the cross-sectional design, which renders causality not possible.

In conclusion, PSBB is both individually and cumulatively associated with HRQOL. As such, strategies to promote behavioral, biological and psychological health, particularly while considering SES, is of major importance. Future work employing a prospective cohort study design would complement the present study’s findings. Ultimately, physicians and others in the field of health and wellness are encouraged to promote holistic health practices that may beneficially affect both psychological and biological parameters that may positively influence an individual’s perceived quality of life.

## Ethical approval

This study was approved by the ethics committee at the National Center for Health Statistics.

## Competing interests

We declare no conflicts of interest.

## Authors’ contributions

All authors were involved in the conceptualization of the study, revising the manuscript and interpreting the results. PDL computed the analyses.

## Acknowledgments

No funding was used to prepare this manuscript.

**Table 1:** Weighted characteristics of the study variables, 2005-2006 NHANES (N = 2524)

**Variable**	**Point estimate**	**95% CI**
Age, mean years	46.2	44.5-47.9
Female, %	50.8	
PSBB index, mean	9.5	9.2-9.7
HRQOL, mean	0.35	0.31-0.39
HRQOL, % score		
0	76.3	
1	15.7	
2	4.4	
3	2.7	
4	0.9	
Biological index, mean	3.1	3.0-3.3
Biological index, % score		
0	1.1	
1	7.9	
2	20.4	
3	30.4	
4	24.6	
5	15.6	
Behavioral index, mean	2.1	2.0-2.3
Behavioral index, % score		
0	5.1	
1	22.2	
2	33.1	
3	29.0	
4	10.5	
Socioeconomic index, mean	3.1	3.0-3.3
Socioeconomic index, % score		
0	0.7	
1	4.6	
2	15.2	
3	33.5	
4	46.0	
Depression, %	4.7	

Abbreviations: PSBB, psycho-socioeconomic bio-behavioral; HRQOL, health-related quality of life.

**Table 2:** Weighted multivariable ordinal regression results evaluating the associations between PSBB and health-related quality of life, 2005-2006 NHANES (N = 2524)

**Model 1**	**β**	**95% CI**	***P*** ** value**
PSBB Index, 1 index score increase	-0.35	-0.44, -0.25	<0.001
**Model 2**			
Behavioral index, 1 index score increase	-0.23	-0.37, -0.09	0.003
Not depressed vs. depressed	-2.99	-3.41, -2.57	<0.001
Socioeconomic index, 1 index score increase	-0.37	-0.48, -0.26	<0.001
Biological index, 1 index score increase	-0.24	-0.37, -0.11	0.001

Abbreviation: PSBB, psycho-socioeconomic bio-behavioral. Both models were adjusted for age and gender.
